# Methylseleninic Acid Induces Lipid Peroxidation and Radiation Sensitivity in Head and Neck Cancer Cells

**DOI:** 10.3390/ijms20010225

**Published:** 2019-01-08

**Authors:** John T. Lafin, Ehab H. Sarsour, Amanda L. Kalen, Brett A. Wagner, Garry R. Buettner, Prabhat C. Goswami

**Affiliations:** 1 Department of Urology, University of Texas Southwestern Medical Center, Dallas, TX 75235, USA; john.lafin@utsouthwestern.edu; 2 The University of Iowa Free Radical and Radiation Biology Program, Department of Radiation Oncology, University of Iowa, Iowa City, IA 52242, USA; ehab-sarsour@uiowa.edu (E.H.S.); amanda-kalen@uiowa.edu (A.L.K.); brett-wagner@uiowa.edu (B.A.W.); garry-buettner@uiowa.edu (G.R.B.)

**Keywords:** head and neck cancer, selenium, methylseleninic acid, radiation, lipid peroxidation, glutathione, tocopherol

## Abstract

Combination radiation and chemotherapy are commonly used to treat locoregionally advanced head and neck squamous cell carcinoma (HNSCC). Aggressive dosing of these therapies is significantly hampered by side effects due to normal tissue toxicity. Selenium represents an adjuvant that selectively sensitizes cancer cells to these treatments modalities, potentially by inducing lipid peroxidation (LPO). This study investigated whether one such selenium compound, methylseleninic acid (MSA), induces LPO and radiation sensitivity in HNSCC cells. Results from 4,4-difluoro-4-bora-3a,4a-diaza-*S*-indacene (BODIPY) C11 oxidation and ferric thiocyanate assays revealed that MSA induced LPO in cells rapidly and persistently. Propidium iodide (PI) exclusion assay found that MSA was more toxic to cancer cells than other related selenium compounds; this toxicity was abrogated by treatment with α-tocopherol, an LPO inhibitor. MSA exhibited no toxicity to normal fibroblasts at similar doses. MSA also sensitized HNSCC cells to radiation as determined by clonogenic assay. Intracellular glutathione in cancer cells was depleted following MSA treatment, and supplementation of the intracellular glutathione pool with *N*-acetylcysteine sensitized cells to MSA. The addition of MSA to a cell-free solution of glutathione resulted in an increase in oxygen consumption, which was abrogated by catalase, suggesting the formation of H_2_O_2_. Results from this study identify MSA as an inducer of LPO, and reveal its capability to sensitize HNSCC to radiation. MSA may represent a potent adjuvant to radiation therapy in HNSCC.

## 1. Introduction

Head and neck squamous cell carcinoma (HNSCC) is a diverse group of cancers that originate from the mouth, nose, throat, or other nearby areas. Over 50,000 new cases of HNSCC are anticipated to arise in the US in 2018; the five-year survival rate is ~64% [1]. Locoregionally advanced HNSCC is often treated with combination radio- and chemotherapy. However, side effects from primary therapy can be debilitating. Radiation and chemotherapy can result in oral mucositis, which can significantly reduce tolerable doses [2]. Even with these aggressive therapy options, about 40% of HNSCC deaths will occur due to the development of therapy resistance [3]. Additional options to sensitize HNSCC cells to current therapies are sorely needed.

Selenium administration shows great promise as a sensitizer to radio- and chemotherapy. Sodium selenite, an inorganic selenium derivative, induces toxicity and radiation sensitization in various cancer cell types with limited effects on normal fibroblasts [4,5,6]. A recent metastudy reported that selenite supplementation in patients receiving radiation therapy reduced deleterious side effects with no protective effects noted in tumors, supporting the use of selenium as an adjuvant to therapy [7]. These studies and others suggest that selenium may sensitize tumors to intervention, while potentially protecting normal tissue. Unfortunately, sodium selenite exhibits toxicity at relatively low doses, with a reported maximum tolerable dose of 10.2 mg m^−2^ [8]. Organoselenium derivatives, such as selenomethionine (SLM) and methylselenocysteine (MSC), are much less toxic than their inorganic counterparts while maintaining the selective effects noted with selenite [9,10]. Organoselenium derivatives exert their anticancer activities through the formation of a common active metabolite, methylselenol [11,12]. SLM and MSC require the action of specific lyase enzymes, such as methionine gamma-lyase (MGL), to release methylselenol [13]. MGL expression is reported to decline in a number of cancer types, suggesting that the formation of methylselenol in tumor tissue by SLM may be slow, limiting its efficacy [14,15,16,17].

Methylseleninic acid (MSA) is an organoselenium derivative that generates methylselenol through its spontaneous reaction with free thiols, such as glutathione [18]. Because the activity of MSA is not reliant upon the expression of lyases, such as MGL, it may represent a more effective antitumor agent than other organoselenium compounds. Previous studies have reported that MSA more effectively reduces TM2H and TM12 hyperplastic mammary cell accumulation than MSC, even at 10-fold lower doses [12]. Additionally, oral administration of MSA reduced the size of PC-3 xenografts in mice by approximately 40%, while administration of SLM, MSC, or sodium selenite exhibited no effects, indicating that MSA is more effective both in vitro and in vivo than other selenium compounds [19]. A combination of MSA and paclitaxel reduced the size of MDA-MB-231 xenografts in mice by about 50%, compared to paclitaxel alone, suggesting that MSA may be an effective adjuvant to current therapies [20]. In these studies, no change in body weight was observed, suggesting that MSA was well tolerated.

Although methylselenol has been identified as the active antitumor metabolite of organoselenium compounds, the mechanism of action following its generation is poorly understood. The combination of MSA and glutathione has been demonstrated to increase lucigenin-based chemiluminescence, which was abrogated by the presence of superoxide dismutase, suggesting the generation of superoxide (O_2_^●−^) [18]. Superoxide may be protonated to form hydroperoxyl radical (HO_2_^●^) or dismutated to form hydrogen peroxide (H_2_O_2_), both of which may contribute to the initiation of lipid peroxidation (LPO) [21,22]. Selenium administration has been associated with elevated markers of LPO, suggesting that these initiating effects occur in vivo [23,24,25]. Because end products of LPO can be highly toxic, MSA-generated methylselenol could, therefore, exhibit toxicity through the superoxide-mediated initiation of LPO. Furthermore, HNSCC patients exhibit higher levels of plasma markers of LPO than matched healthy subjects, suggesting that HNSCC may be particularly susceptible to LPO [26,27].

Results presented herein reveal that MSA exhibits toxicity and radiation sensitization of Cal27 and SCC25 HNSCC cells. Cal27 cells were found to be much more sensitive to MSA compared to SLM or MSC, while normal human fibroblasts were resistant to MSA-induced toxicity. Initiation of oxidative distress via lipid peroxidation appears to be the underlying mechanism for toxicity. Our data suggest that the toxic effects of MSA are mediated by a glutathione-dependent formation of an initiator of LPO. MSA may be a useful adjuvant to radiation therapy.

## 2. Results

### 2.1. MSA is More Toxic to HNSCC Cells than Other Organoselenium Derivatives, and Causes Cell Death in a Dose- and Time-Dependent Manner

**Figure 1:**
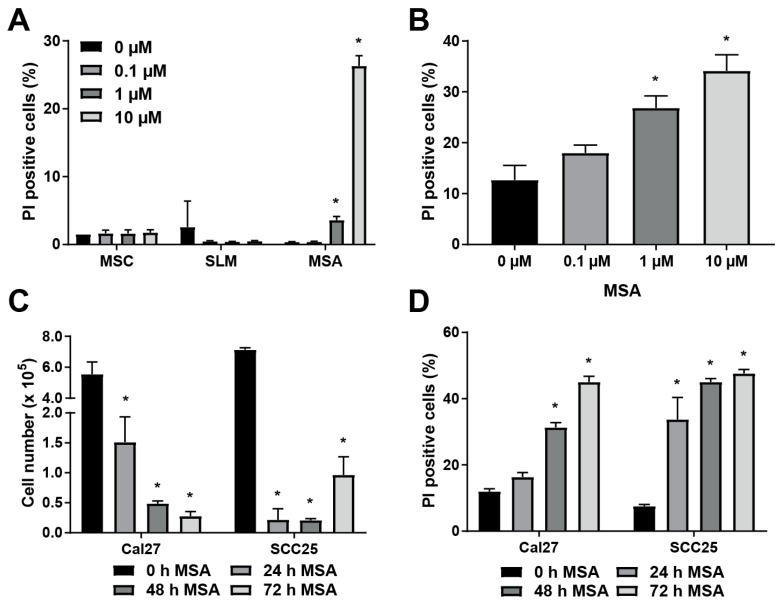
Methylseleninic acid (MSA) is toxic to Cal27 and SCC25 HNSCC cells in a dose- and time-dependent manner. (**A**) PI exclusion assay of Cal27 cells treated with the shown concentrations of Se-methylselenocysteine (MSC), seleno-l-methionine (SLM), or MSA for 72 h. (**B**) Propidium iodide (PI) exclusion assay of SCC25 cells treated with 0 to 10 µM MSA for 72 h. (**C**) Cell counts of Cal27 and SCC25 cells following treatment with 10 µM MSA for 0 to 72 h. (**D**) PI exclusion assay of Cal27 and SCC25 cells after treatment with 10 µM MSA for 0 to 72 h. *, statistical significance relative to 0 µM MSA controls; *p* < 0.05, *n* = 3.

Organoselenium derivatives SLM and MSC require enzymatic action to generate methylselenol, the common active metabolite. Therefore, methylselenol generation from SLM and MSC will be less in situations where these enzymes are poorly expressed. MSA requires no enzymatic activity to generate methylselenol [18]. To determine if MSA exhibits greater toxicity than other organoselenium compounds, Cal27 cells were treated with MSA, MSC, or SLM for 72 h, and viability was assessed with propidium iodide (PI) exclusion assay. MSC and SLM exhibited no toxicity at doses up to 10 µM, Figure 1A. Treatment with 1 µM MSA resulted in a small but significant increase in PI positive cells, while 10 µM MSA resulted in about 30% PI-positive cells. To ensure that MSA toxicity was not cell-line dependent, the effects of MSA treatment on SCC25 cells were also examined. A dose-dependent increase in PI positive SCC25 cells was observed with MSA treatment, with approximately 30% of the cells staining positive at 10 µM, Figure 1B. These results indicate that Cal27 cells are more sensitive to MSA than other organoselenium derivatives and that MSA-induced toxicity is dose-dependent in both Cal27 and SCC25 cell lines.

The sensitizing effects of organoselenium compounds on cancer cells have been reported to occur as early as 24 h following the beginning of treatment [28]. To determine the temporal aspects of the toxicity resulting from exposure to MSA, Cal27 and SCC25 cells were treated with MSA for varying durations and the toxicity was examined by measuring changes in cell numbers as well as flow cytometry measurements of the percentage of PI-positive (non-viable) and PI-negative (viable) cell populations. SCC25 and Cal27 cells both showed a marked decline in cell number as early as 24 h after initiation of treatment with MSA, with a reduction in cell number of about 75% and 95% in Cal27 and SCC25, respectively, Figure 1C. Cal27 cell numbers continued to decline up to 72 h, while SCC25 cell number appeared to begin to recover at 72 h. The rapid onset of a reduction in cell number correlated with an increase in the percentage of PI-positive Cal 27 cells at 48 h of treatment, Figure 1D. SCC25 cells exhibited significant toxicity as early as 24 h. SCC25 maximal toxicity (45% PI-positive cells) was reached by 48 h in the period examined, while Cal27 reached similar levels at 72 h. Together, these results indicate that the MSA treatment exhibits greater toxicity to HNSCC than treatments with MSC and SLM and that this toxicity is dose- and time-dependent. Furthermore, treatment with MSA appears to be more toxic to SCC25 compared to Cal27 cells.

### 2.2. MSA Treatment Sensitizes HNSCC Cells to Radiation

**Figure 2:**
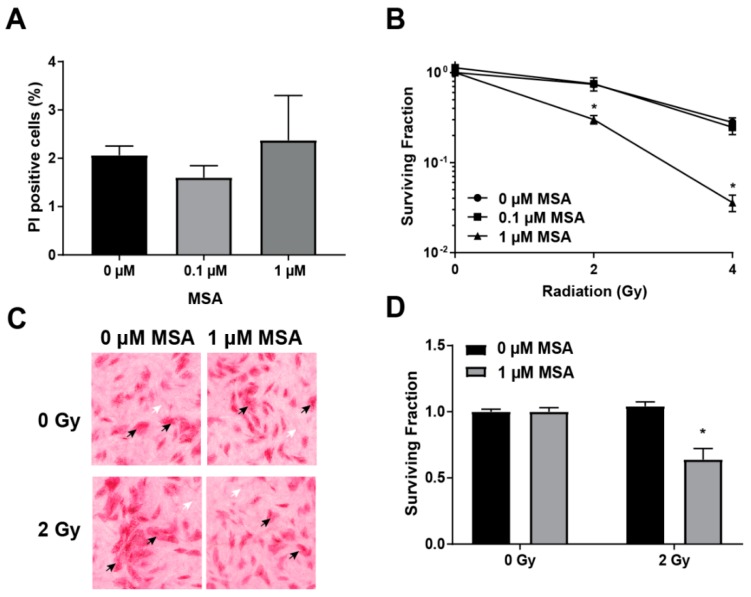
MSA selectively sensitizes head and neck squamous cell carcinoma (HNSCC) cells to radiation. (**A**) PI exclusion assay of normal human fibroblasts (NHF) treated with MSA 24 h. (**B**) Clonogenic assay of Cal27 cells treated with MSA 48 h before irradiation with γ-rays. (**C**) Representative images of Cal27 cells in co-cultures with NHF that were treated with MSA 48 h before irradiation with γ-rays. Black arrows: Cal27 colonies; white arrows: quiescent NHF. (**D**) Quantitation of Cal27 clonogenic survival in co-cultures of Cal27 and NHF that were treated with MSA 48 h before irradiation with γ-rays. *, statistical significance relative to 0 µM MSA controls; *p* < 0.05, *n* = 3.

Selenium compounds, such as sodium selenite and seleno-l-methionine, sensitize cancer cells to radiation [4,5,10,29]. Furthermore, this sensitization is frequently noted to be selective for cancer cells [29]. Fibroblasts are often thought to make up the majority of the non-cancer cellular fraction in the tumor stroma [30,31]. To determine if normal human fibroblasts (NHF) were resistant to MSA toxicity, a PI exclusion assay was utilized. PI-positive (non-viable) NHF population did not increase following MSA treatment, Figure 2A. MSA (1 µM) treatment more than doubled non-viable Cal27 and SCC25 populations, Figure 1A,B, demonstrating the selective effects of MSA to HNSCC over NHF. To determine if MSA sensitizes HNSCC to radiation, Cal27 cells were treated with MSA for 48 h before 2 or 4 Gy irradiation, and toxicity was analyzed by using a clonogenic assay. Irradiated cells without MSA treatment showed a surviving fraction of 0.75 and 0.28 at 2 and 4 Gy, respectively, Figure 2B. Treatment with 0.1 µM MSA did not significantly alter surviving fraction of Cal27 cells: 0.66 and 0.22 at 2 and 4 Gy, respectively. Interestingly, prior treatment with 1 µM MSA significantly reduced the surviving fraction to 0.3 and 0.03 at 2 and 4 Gy compared to a surviving fraction of 0.75 and 0.28 without MSA treatment.

Radiation response is frequently dependent upon the support of the tumor stroma. To determine if the tumor stroma impacts the ability of MSA to sensitize Cal27 cells to radiation, a co-culture clonogenic assay was utilized. Cal27 cells were plated on lawns of quiescent normal human fibroblasts (NHF), and co-cultures were treated with 1 μM MSA for 48 h before irradiation. Even with NHF present, MSA treatment resulted in a 40% decline of surviving fraction following 2 Gy radiation, Figure 2D. Additionally, the lawn of NHF was not disturbed by MSA, further indicating that MSA was not toxic to NHF even in combination with radiation, Figure 2C. These results indicate that MSA treatment potently and selectively sensitizes Cal27 cells to radiation in co-cultures of NHF.

### 2.3. MSA Treatment Induces Lipid Peroxidation in HNSCC Cells

**Figure 3:**
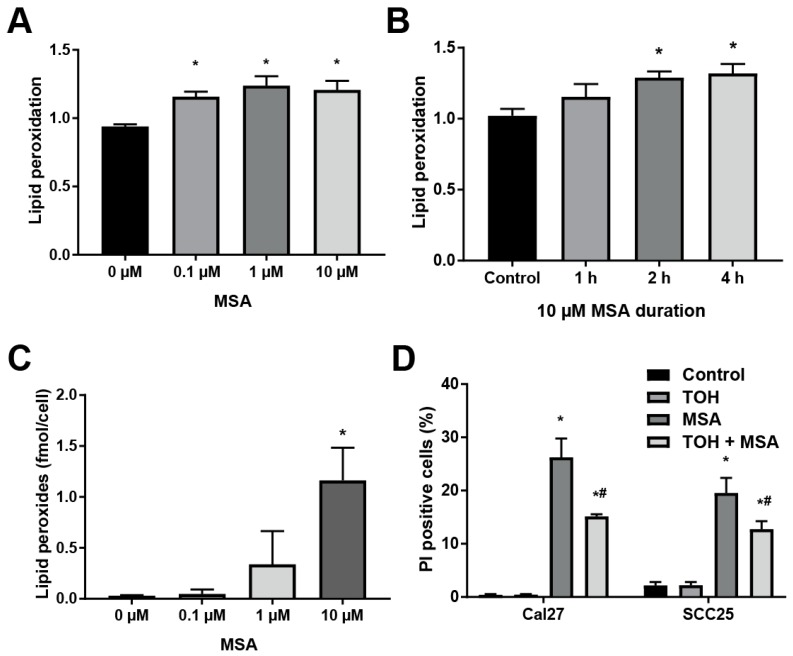
MSA induces lipid peroxidation in HNSCC cells. (**A**) Lipid peroxidation in Cal27 cells assessed by 4,4-difluoro-4-bora-3a,4a-diaza-*S*-indacene (BODIPY) C-11 staining following 72 h treatment with 0 to 10 µM MSA. (**B**) Lipid peroxidation in Cal27 cells following treatment with 10 μM MSA. (**C**) Lipid peroxides in Cal27 cells as assessed by the Cayman Chemical LPO Kit following treatment with 0 to 10 µM MSA for 72 h. (**D**) PI exclusion assay of Cal27 and SCC25 cells treated with 20 µM α-tocopherol acetate (TOH) for 24 h, 10 µM MSA for 72 h, or pre-treatment with TOH for 24 h followed by treatment with MSA. *, statistical significance relative to 0 µM MSA controls; #, statistical significance relative to MSA alone; *p* < 0.05, *n* = 3.

Organoselenium compounds are theorized to be metabolized through a multitude of pathways to a central active metabolite, methylselenol, which exerts toxicity. Due to the highly reactive nature of methylselenol, studies concerning its mechanism of toxicity are sorely lacking. However, markers of lipid peroxidation have been found to rise in patients treated with selenium [24], suggesting that high dose selenium may induce lipid peroxidation. To determine if MSA treatment induces lipid peroxidation in HNSCC cells, MSA-treated Cal27 cells were labeled with the dye BODIPY C-11. This dye integrates into membranes and emits maximally at 590 nm. Upon oxidation by an initiator or propagator of lipid peroxidation, the maximal emission shifts to 510 nm. By reading both channels simultaneously, a ratio of oxidized to reduced dye can be calculated, providing a snapshot of lipid peroxidation initiation and propagation. Lipid peroxidation was found to be up to 30% elevated in Cal27 cells treated with MSA for 72 h, Figure 3A. An elevation was noted at a dose as low as 0.1 µM MSA, suggesting a powerful potential for initiation. Examination of lipid peroxidation at very early time intervals indicated that lipid peroxidation was initiated as early as 2 h, and maintained at a relatively stable level up to 72 h, Figure 3B. To determine if these increases in initiation and propagation events resulted in elevated lipid hydroperoxides, the Cayman Lipid Hydroperoxide Assay kit was utilized. Cal27 cells treated with 10 µM MSA for 72 h were found to have 1.16 fmol lipid hydroperoxide per cell, nearly 40 times as much as untreated cells, Figure 3C. These data indicate that treatment with MSA induces lipid peroxidation potently and persistently and that this induction results in a significant accumulation of lipid hydroperoxides.

Lipid peroxidation is a deleterious oxidative chain reaction that can form toxic products, such as MDA or 4-HNE. Induction of uncontrolled lipid peroxidation can damage critical biomolecules resulting in cell death. To determine if MSA-induced toxicity is caused by lipid peroxidation, Cal27 and SCC25 cells were pre-treated with a lipid peroxidation chain terminator, α-tocopherol acetate (TOH), before MSA treatment. Treatment with TOH alone did not impact the percentage of PI-positive cell populations in either cell line, Figure 3D. However, treatment with TOH before MSA treatment reduced the percentage of the PI-positive cell population in Cal27 from 26% to 15%, and in SCC25 from 20% to 12%, an approximate 40% decline in the PI-positive populations. These results show that lipid peroxidation is an essential step in MSA-induced toxicity of HNSCC cells.

### 2.4. N-Acetyl-l-Cysteine Exacerbates MSA Toxicity in HNSCC

**Figure 4:**
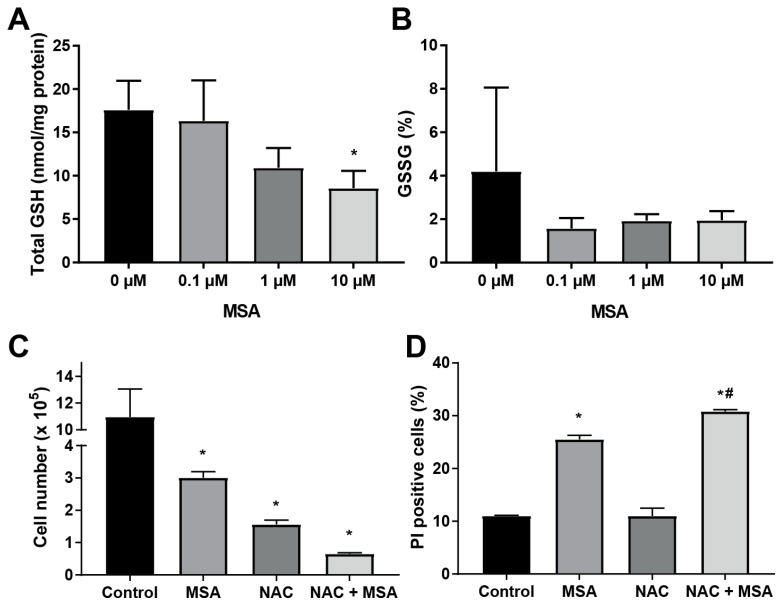
MSA exhibits toxicity in a glutathione (GSH)-dependent manner. (**A**) Total GSH in Cal27 cells treated with 0 to 10 µM MSA for 72 h. (**B**) Percent of GSH existing as glutathione disulfide (GSSG) in Cal27 cells treated with 0 to 10 µM MSA for 72 h. (**C**) Cell counts of Cal27 cells following treatment with 5 mM *N*-Acetyl-l-Cysteine (NAC) for 24 h, and/or 10 µM MSA for 72 h. The NAC + MSA group received 5 mM NAC for 24 h before treatment with MSA. (**D**) PI exclusion assay of Cal27 cells treated with 5 mM NAC for 24 h, and/or 10 µM MSA for 72 h. The NAC + MSA group received 5 mM NAC for 24 h before treatment with MSA. *, statistical significance relative to 0 µM MSA controls; #, statistical significance relative to NAC alone; *p* < 0.05, *n* = 3.

MSA spontaneously reacts with glutathione (GSH) to form its active metabolite, methylselenol, and GSH disulfide (GSSG), see below [18]. As the principal intracellular redox buffer, GSH is critical to normal cellular function. GSSG may be cytotoxic; it can be recycled to GSH by glutathione reductase, exported from the cell, or form mixed protein disulfides. To determine if MSA treatment influences GSH levels in HNSCC cells, the intracellular GSH content was measured in MSA-treated Cal27 cells using a biochemical assay [32]. Results indicate a dose-dependent decrease of total GSH in MSA-treated Cal27 cells, Figure 4A. Untreated Cal27 cells exhibited a total GSH concentration of about 17 nmol (mg protein)^−1^. Treatment with MSA lowered this to below 10 nmol (mg protein)^−1^, a 40% decline. Despite this marked decline, intracellular GSSG was not found to increase following MSA treatment, but rather also declined, Figure 4B. The decline in GSH levels suggests that GSH may have a significant role in the MSA-induced toxicity of HNSCC. This premise is further supported by results showing *N*-Acetyl-l-Cysteine (NAC) treatments exacerbating MSA-induced toxicity in Cal27 cells, Figure 4C. NAC is a membrane permeable precursor to GSH, stimulating its production; its effects are detectable within 4 h [33,34]. Cal27 cells were treated with 5 mM NAC for 24 h, washed, and treated with 10 µM MSA for 72 h. Cell number declined from 3 × 10^5^ in cultures treated with MSA alone, to 0.5 × 10^5^ cells, Figure 4C. A PI exclusion assay revealed that the combination of NAC and MSA resulted in a 30% non-viable cell population, while MSA alone resulted in only 25%, Figure 4D. Although NAC alone reduced cell number from 11 × 10^5^ to 0.6 × 10^5^, it had no effect on viability. This is consistent with prior reports from our lab that NAC treatment induces a cell cycle arrest [33,34]. These results indicate that GSH facilitates MSA-induced free radical chemistry (see below), leading to lipid peroxidation and HNSCC cytotoxicity.

### 2.5. MSA Treatment Enhances GSH-Dependent O_2_ Consumption

**Figure 5:**
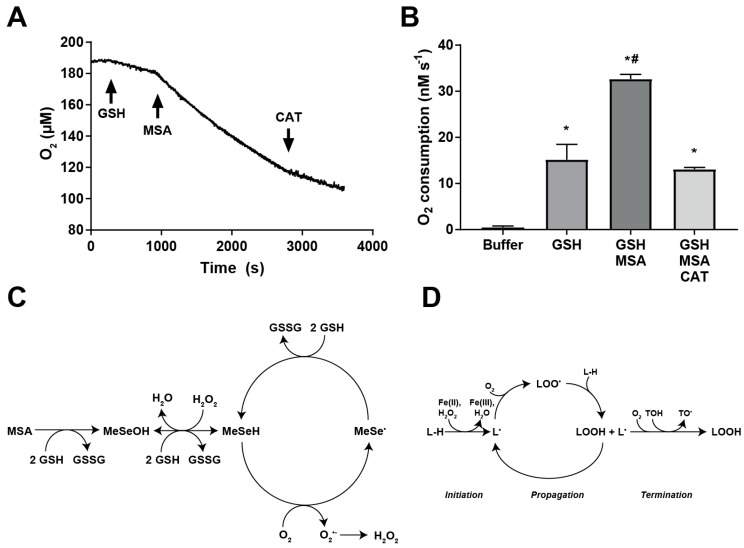
MSA enhances the consumption of O_2_ by glutathione. (**A**) Representative trace of O_2_ concentration in 3.00 mL pH 9.2 50 mM borate buffer at 37 °C. GSH and MSA added to a final concentration of 2 mM and 250 µM, respectively, at indicated time points. (**B**) Rate of O_2_ consumption in 3.00 mL pH 9.2, 50 mM borate buffer at 37 °C. (**C**) Schematic of metabolism of MSA. MSA is reduced by GSH to methylselenol (MeSeH) through a methylselenenic acid (MeSeOH) intermediate. MeSeH may cycle with O_2_ and GSH through a methylselenyl radical intermediate (MeSe^●^) to generate H_2_O_2_, potentially through an O_2_^●−^ intermediate. MeSeH may also exhibit peroxidase activity, consuming H_2_O_2_. (**D**) Schematic of the process of lipid peroxidation. The process is initiated by abstraction of a hydrogen atom from a lipid, forming a carbon-centered lipid radical (L^●^). The reaction is propagated by the addition of O_2_, followed by abstraction of another hydrogen atom from a neighboring lipid, forming LOOH and a new L^●^. The chain can be terminated by a donor antioxidant, such as tocopherol (TOH). The resulting tocopheroxyl radical (TO^●^) radical does not efficiently further oxidize lipids. *, statistical significance relative to buffer alone; #, statistical significance to GSH alone; *p* < 0.05, *n* = 3.

MSA can be reduced by free thiols, such as GSH, to form its active metabolite, methylselenol, Figure 5C. Because the p*K*_a_ of the selenohydryl group is 5.2, it primarily exists in biological systems as its conjugate base, the highly reactive methylselenolate anion (MeSe^−^) [35]. This reactive species may initiate a cyclic reaction with molecular oxygen and GSH to cycle between a methylselenyl radical intermediate and methylselenolate anion, forming O_2_^●−^ (which is rapidly dismuted to H_2_O_2_) and GSH to GSSG as products. This cyclic reaction is anticipated to account for the toxicity of organoselenium compounds [18,36]. In support of this chemistry, a previous study demonstrated the involvement of oxygen to the cyclic reaction between a related compound, selenocystamine, and GSH [36]. To determine if MSA may also undergo a similar reaction, O_2_ consumption was monitored in a cell-free system containing MSA and GSH, Figure 5A. This system was held at pH 9.2, as this is the reported optimum pH for selenium-catalyzed O_2_^●−^ generation [18]. O_2_ was observed to disappear from the buffer at a rate of approximately 3 nM s^−1^. Addition of MSA to buffer did not change the rate of consumption of O_2_ (data not shown). Addition of GSH in the absence of MSA resulted in the disappearance of O_2_ at a rate of approximately 15 nM s^−1^, Figure 5B. Interestingly, the addition of MSA (250 µM) following GSH doubled the rate of O_2_ consumption to approximately 32 nM s^−1^. Furthermore, the addition of 500 IU catalase caused the rate of O_2_ consumption to return to 13 nM s^−1^, approximately the same as GSH alone. These results indicate an O_2_-dependent reaction occurring between MSA and GSH that may result in the formation, but not accumulation, of H_2_O_2_. H_2_O_2_ may also contribute to another O_2_-dependent reaction between GSH and MSA. The resulting flux of H_2_O_2_ may facilitate lipid peroxidation through the iron-dependent generation of HO^●^, resulting in MSA-induced toxicity of HNSCC. Previous reports indicate elevated markers of LPO in HNSCC patients [26,27], suggesting that MSA-induced selective cytotoxicity may be due to higher baseline levels of LPO in HNSCC compared to normal cells.

## 3. Discussion

Results presented here show that MSA treatment results in toxicity and enhanced radiation sensitivity in HNSCC cells and that this toxicity may be facilitated by glutathione and oxygen-mediated reactions resulting in toxicity. BODIPY C-11 oxidation data indicate that lipid peroxidation is associated with this oxidative process, can be detected within 2 h of the initiation of MSA treatment, and persists for at least 72 h. Lipid hydroperoxides were detected in a dose-dependent manner following treatment with MSA, consistent with a role for lipid peroxidation and corroborating BODIPY C-11 oxidation experiments. In a cell-free system, MSA doubled the rate of GSH-dependent O_2_ consumption. Consistent with results from the cell-free experiments, intracellular GSH levels decline in Cal27 cells following exposure to MSA. Supplementation of the intracellular GSH pool by pre-treatment with NAC further sensitized, rather than protected, the cells from MSA-induced toxicity. These results suggest that MSA reacts with intracellular GSH, yielding reactive oxygen species capable of inducing lipid peroxidation and cell death.

MSA exhibited significant toxicity to Cal27 and SCC25 HNSCC cells, Figure 1. Similar organoselenium compounds SLM and MSC did not show any toxicity at doses up to 10 μM, while 10 μM MSA resulted in a nearly 30% PI-positive (non-viable) population of cells, Figure 1A. The active metabolite of all three of the examined compounds is methylselenol [11,12]. SLM and MSC release methylselenol following processing by lyase enzymes, such as MGL [13]. Several reports have identified MGL as a tumor suppressor gene, suggesting that its expression is reduced in tumors [14,15,16,17]. Overexpression of MGL in ovarian cancer cells resulted in up to 1000-fold sensitization to SLM [37]. Furthermore, hepatoma xenografts treated with the combination of adenovirus-delivered MGL and SLM (1 µmol d^−1^, IP) exhibited a drastic decline in tumor size compared to SLM alone, indicating the necessity of MGL for SLM to generate methylselenol [37]. MSA generates methylselenol by a direct and spontaneous reaction with GSH, obviating the need for MGL, Figure 5C [13,18]. Previous studies have identified MSA as more effective than MSC at inducing apoptosis and inhibiting cell growth in murine mammary cell cultures [12]. MSA also more effectively reduced prostate cancer xenograft size than SLM or MSC with no change in body weight [19]. Our results indicate that HNSCC is also more sensitive to MSA than SLM or MSC. MSA was also found to render Cal27 HNSCC cells sensitive to radiation, Figure 2B. Although other selenium compounds have been reported to induce sensitivity of cancer cells to radiation, the ability of MSA to do so has not yet been reported [4,10]. The increased toxicity of MSA compared to other selenium compounds suggests that it may also more effectively sensitize cancer cells to radiation. Additionally, the cytotoxic and sensitizing effects appear to be selective to cancer cells, as NHF were relatively resistant to MSA-induced toxicity, Figure 2A, and quiescent lawns of NHF were undisturbed by a combination of MSA and radiation, Figure 2C.

MSA treatment increased BODIPY C11 oxidation in Cal27 cells, Figure 3A,B. BODIPY C11 may be oxidized by LPO initiators, such as hydroperoxyl radical, and propagators, such as lipid peroxyl radical [38]. The dye is insensitive to LOOH and aldehydic end products of LPO, such as malondialdehyde (MDA) or 4-hydroxynonenal (4-HNE). A dose-dependent increase of BODIPY C11 oxidation was not observed at 72 h of MSA treatment. However, MSA treatment did result in a dose-dependent increase in the accumulation of LOOH, as determined by the Cayman LPO Kit, Figure 3C. Additionally, BODIPY C11 dye oxidation was found to stabilize as early as 2 h of MSA treatment, Figure 3B. These results suggest that MSA induces LPO rapidly and persistently and that this results in an accumulation of toxic lipid hydroperoxides. Furthermore, LPO appears essential to the toxicity of MSA, as pre-treatment with α-tocopherol acetate, an inhibitor of LPO, protected the cells from MSA-induced toxicity, Figure 3D. Previous studies have mainly focused on selenium administration as an inhibitor of LPO, presumably due to the induction of the glutathione peroxidase system [39]. However, evidence exists to suggest that selenium may initiate rather than inhibit LPO. Pre-treatment with sodium selenite (single dose 2 mg kg^−1^, IP) in a murine model of heavy metal poisoning found a 4-fold increase of liver MDA content over metal alone [23]. Administration of ebselen, a synthetic organoselenium compound, increased liver MDA content in rats by approximately 20% [25]. Serum MDA levels were elevated in ovarian cancer patients following the administration of 50 μg of selenium as selenized yeast (4.8 μM compared to 3.9 μM) [24]. These reports and others suggest that in some circumstances, selenium administration may induce LPO, although the mechanisms are yet unclear. Because MSA more effectively generates methylselenol, the active metabolite, than similar selenium compounds, it may also more potently induce LPO.

MSA treatment of Cal27 cells resulted in a depletion of total intracellular GSH content, Figure 4A,B. MSA has been reported to deplete GSH in A549 lung cancer and HepG2 hepatoma cells [40,41]. In HepG2 cells, a biphasic response was noted: A 10 µM treatment of MSA for 24 h caused intracellular GSH to increase by about 75%, while 25 µM MSA resulted in an approximate 20% decline in GSH, with no change in GSSG. This biphasic response was not noted in Cal27 cells; total GSH content was unchanged or declined at all doses tested, Figure 3A. Furthermore, treatment with MSA (10 µM) for 24 h decreased cell numbers of Cal27 and SCC25; a significant increase in the non-viable population, Figure 1C,D. Apoptosis of HepG2 cells as detected by LDH was not noted at 10 µM MSA for 24 h, suggesting that HepG2 cells are more resistant to MSA than HNSCC [41]. This concentration coincides with elevated GSH in HepG2, which suggests that the biphasic response may play a role in resistance of MSA. Interestingly, despite a decline in intracellular GSH levels following MSA treatment, no change was noted in GSSG, Figure 4B. This may be due to increased GSSG efflux through membrane transporters, such as MRP1 [42]. GSH may also be consumed without GSSG formation through conjugation, suggesting that MSA treatment may induce increased GSH conjugation forming mixed disulfides. Supplementation of the intracellular thiol pool by pre-treatment with NAC sensitized Cal27 cells to MSA, Figure 4C,D. Similar results were reported with MSA treatment of HepG2 cells [41]. These results suggest that a reaction between MSA and a thiol, such as GSH, is essential for MSA-induced cytotoxicity.

Oxygen consumption by GSH was doubled in the presence of MSA in a cell-free system, Figure 5A,B. Furthermore, the addition of catalase to this system returned the rate of O_2_ consumption to those similar to GSH alone. These results suggest the formation, but not accumulation, of H_2_O_2_. Had H_2_O_2_ accumulated, the addition of catalase would have returned O_2_ to the system, i.e., an increase in the concentration of O_2_ would have been observed. Selenol species may exhibit peroxidase activity, as evidenced by the active site selenol of selenium-containing glutathione peroxidases [36]. Methylselenol may, therefore, exhibit peroxidase activity, yielding a methylselenenic acid, which may again react with GSH to regenerate methylselenol, Figure 5C. Previous reports indicate the potential for the formation of O_2_^●−^ in systems containing selenium and GSH. The addition of MSA to a solution of GSH is reported to increase lucigenin-based chemiluminescence [13,18]. This effect was abrogated by the presence of superoxide dismutase, suggesting the formation of O_2_^●−^. Additionally, an examination of the reaction kinetics of GSH and selenocystamine, an organoselenium compound similar to MSA, suggests a cyclic reaction yielding superoxide [36]. Following O_2_^●−^ generation, methylselenol may be regenerated by an additional reduction by GSH, yielding a cyclic reaction capable of generating large amounts of O_2_^●−^ [18,36]. These reactions are summarized in Figure 5C. Following its formation, O_2_^●−^ can dismute to H_2_O_2_, either spontaneously or through the action of superoxide dismutase [43]. H_2_O_2_ may contribute to LPO initiation through the generation of hydroxyl radical by iron-mediated Fenton chemistry [44,45]. Superoxide can also be protonated to form hydroperoxyl radical, which is a powerful LPO initiator [21,22]. The contribution of O_2_^●−^ to selenium-based cytotoxicity has been further demonstrated by the protective effects of a superoxide dismutase mimetic [46]. Methylselenol may, therefore, initiate lipid peroxidation chain reactions through superoxide-mediated products.

Overall, results from this study indicate that MSA sensitizes HNSCC cell to radiation and exhibits toxicity through a GSH-dependent induction of LPO. LPO occurs more readily in cells with higher polyunsaturated fatty acid content [47]. Many types of cancer exhibit greater lipid content than their respective normal counterparts, including colon, prostate, pancreatic, and clear cell renal carcinoma [48,49,50,51,52]. MSA may, therefore, exhibit selective cytotoxicity in cancer cells on the basis of altered lipid content. A pre-clinical study examining the efficacy of MSA (4 mg kg^-1^ d^−1^, PO) in treating prostate cancer xenografts found a nearly 40% reduction in tumor size following MSA treatment, with no change in body weight [19]. Similarly, MSA (4.5 mg kg^−1^ d^−1^, PO) combined with paclitaxel (10 mg kg^−1^ week^−1^, IP) reduced breast cancer xenograft tumor size with no change in body weight [20]. These studies suggest that MSA is selectively toxic to cancer cells. The work presented herein suggests that this selectivity may be in part due to a differential sensitivity to LPO in cancer compared to normal cells.

## 4. Materials and Methods

### 4.1. Cell Culture and Reagents

Head and neck squamous carcinoma cell lines Cal27 (tongue origin, CRL-2095) and SCC25 (tongue origin, CRL-1628) were purchased from ATCC (Manassas, VA, USA). Both lines have mutated p53, are epidermal growth factor receptor (EGFR) positive, and human papillomavirus (HPV) negative. Normal human fibroblasts (NHF) were obtained from the Coriell cell repository (AG01522D). Cells were cultured in Dulbecco’s Modified Eagle’s Medium (DMEM) (Thermo Fisher Scientific, Waltham, MA, USA), supplemented with antibiotics and 10% bovine calf serum (HNSCC cells, Thermo Fisher Scientific, Waltham, MA, USA) or 10% fetal bovine serum (NHF, Sigma-Aldrich, St. Louis, MO, USA). All cells were grown in humidified incubators set to 37 °C, 5% CO_2_, and atmospheric oxygen.

Seleno-l-methionine (SLM, S3132), *N*-acetylcysteine (NAC, A9165), glutathione (GSH, G4251), glutathione disulfide (GSSG, G4376), glutathione reductase (GR, G3664), 2-vinylpyridine (2-VP, 132292), 5,5-dithio-bis-(2-nitrobenzoic acid) (DTNB, D8130), and 5-sulfosalicylic acid (SSA, S2130) were purchased from Sigma-Aldrich. Reduced nicotinamide adenine dinucleotide phosphate (NADPH) (481973) was purchased from EMD Millipore (Burlington, MA, USA). Methyl-Se-selenocysteine (MSC) and methylseleninic acid (MSA) were generous gifts of the laboratory of Youcef Rustum (Roswell Park Cancer Institute, Buffalo, NY, USA). BODIPY 581/591 C-11 (D3861) and CellTracker Green CMFDA (C7025) were purchased from Thermo Fisher Scientific (Waltham, MA, USA). Lipid Hydroperoxide (LPO) Assay Kit (705002) was purchased from Cayman Chemical Company (Ann Arbor, MI, USA).

### 4.2. Irradiation

Exponentially growing cells were irradiated at the Free Radical and Radiation Research Core Facility at The University of Iowa. All irradiated cells received a single dose of γ-rays from a cesium-137 irradiator (JL Shephard, San Fernando, CA, USA) at a dose rate of 0.65 Gy min^−1^. Cell survival was measured using a clonogenic assay following a previously published method [53].

### 4.3. Propidium Iodide Exclusion Assay

Following treatment, cultures were trypsinized, washed, and resuspended in cold phosphate buffered saline (PBS). The suspended cultures were filtered and labeled with 1 μg mL^−1^ propidium iodide for 5 min on ice. Flow cytometry was completed on a Becton-Dickinson FACScan at the University of Iowa Flow Cytometry Core. Data from 10,000 events were collected in list mode. The population of PI-positive (non-viable) and negative (viable) cells were calculated with FlowJo software (FlowJo, LLC, Ashland, Oregon, USA).

### 4.4. BODIPY C-11 Assay

Following MSA treatment, adherent cells were washed and labeled with 5 μM BODIPY C-11 in DMEM lacking serum and antibiotics for 15 min at 37 °C. Following labeling, cultures were collected by trypsinization, washed, resuspended in cold PBS and filtered. Samples were read on a Becton-Dickinson LSR II flow cytometer using channels for Texas Red (reduced dye) and fluorescein isothiocyanate (FITC, oxidized dye) simultaneously at The University of Iowa Flow Cytometry Core. Populations were gated and analyzed with FlowJo software (version 7.6.5), and ratios of oxidized:reduced dye were calculated.

### 4.5. Total Lipid Hydroperoxide Determination

A Cayman Lipid Hydroperoxide Assay Kit was used to determine total lipid hydroperoxides in cell samples. Cal27 cells were treated with MSA for 72 h. Following treatment, cells were collected by trypsinization and counted. Total lipid extracts were obtained and analyzed as recommended by the manufacturer in glass cuvettes on a Beckman DU650 spectrophotometer. Lipid hydroperoxides per cell was quantified by construction of an appropriate standard curve and normalized to cell number, as determined by a Z1 Coulter Counter (Beckman-Coulter, Brea, CA, USA).

### 4.6. Glutathione Determination

Following treatment, cells were collected by trypsinization, washed, and pellets were lysed in ice cold 5% sulfosalicylic acid. Extracts were stored at −80 °C until analysis. Following centrifugation, the supernatant was removed and used for the glutathione assay; protein precipitate was dissolved in 1% SDS, 0.1 M NaOH for protein determination. Total glutathione was determined as described previously on a Beckman DU-650 spectrophotometer [54]. Glutathione disulfide (GSSG) was determined using the method of Griffith and Anderson [32]. Rates of reaction were compared to glutathione or glutathione disulfide standard curves and normalized to protein content as determined by bicinchoninic acid (BCA) (Thermo Fisher Scientific, Manassas, VA, USA).

### 4.7. Oxygen Consumption

Involvement of oxygen in the reactions of glutathione and MSA was investigated by recording oxygen consumption during reaction progress in a cell-free system with an ESA BioStat Multi Electrode System and YSI Oxygen Probe (Yellow Springs Instrument Co., Yellow Springs, OH, USA) at the Free Radical and Radiation Research Core Facility at The University of Iowa. Measurements were conducted in 3.00 mL of 50 mM pH 9.2 borate buffer at 37 °C. Initial oxygen concentration was assumed to be 188 µM, as reported previously [55]. The reactants glutathione, MSA, and catalase were introduced sequentially to final concentrations of 2 mM, 250 µM, and 167 IU mL^−1^, respectively.

### 4.8. Statistical Analysis

Statistical analysis was completed using Prism (GraphPad Software, San Diego, CA, USA). Two-way ANOVA with post-hoc analysis was completed to determine statistical significance. Homogeneity of variance was assumed at a 95% confidence interval. Results from at least three biological replicates with *p* < 0.05 were considered significant.
